# Aptamers and Aptasensors for Highly Specific Recognition and Sensitive Detection of Marine Biotoxins: Recent Advances and Perspectives

**DOI:** 10.3390/toxins10110427

**Published:** 2018-10-25

**Authors:** Lianhui Zhao, Yunfei Huang, Yiyang Dong, Xutiange Han, Sai Wang, Xingguo Liang

**Affiliations:** 1 College of Food Science and Engineering, Ocean University of China, Qingdao 266003, China; zhaolianhuiouc@163.com (L.Z.); huangyunfeiouc@163.com (Y.H.); hanxutiangeouc@163.com (X.H.); liangxg@ouc.edu.cn (X.L.); 2 College of Life Science and Technology, Beijing University of Chemical Technology, Beijing 100029, China; yydong@mail.buct.edu.cn; 3 Laboratory for Marine Drugs and Bioproducts of Qingdao National Laboratory for Marine Science and Technology, Qingdao 266000, China

**Keywords:** marine biotoxin, aptamer, aptasensor, rapid detection, food safety

## Abstract

Marine biotoxins distribute widely, have high toxicity, and can be easily accumulated in water or seafood, exposing a serious threat to consumer health. Achieving specific and sensitive detection is the most effective way to prevent emergent issues caused by marine biotoxins; however, the previous detection methods cannot meet the requirements because of ethical or technical drawbacks. Aptamers, a kind of novel recognition element with high affinity and specificity, can be used to fabricate various aptasensors (aptamer-based biosensors) for sensitive and rapid detection. In recent years, an increasing number of aptamers and aptasensors have greatly promoted the development of marine biotoxins detection. In this review, we summarized the recent aptamer-related advances for marine biotoxins detection and discussed their perspectives. Firstly, we summarized the sequences, selection methods, affinity, secondary structures, and the ion conditions of all aptamers to provide a database-like information; secondly, we summarized the reported aptasensors for marine biotoxins, including principles, detection sensitivity, linear detection range, etc.; thirdly, on the basis of the existing reports and our own research experience, we forecast the development prospects of aptamers and aptasensors for marine biotoxins detection. We hope this review not only provides a comprehensive summary of aptamer selection and aptasensor development for marine biotoxins, but also arouses a broad readership amongst academic researchers and industrial chemists.

## 1. Introduction

Marine biotoxins, also known as algal biotoxins, are a large and diverse group of highly active metabolites from marine organisms, and can be accumulated at a high level in water or seafood via the food chain. More than 1000 kinds of marine biotoxins have been identified, and they are classified into three categories; i.e., polyether toxins, polypeptide toxins, and alkaloid toxins, according to the difference of their chemical structures [1,2,3,4]. For example, okadaic acid (OA) is a typical polyether toxin, and the well-known tetrodotoxin (TTX) is classed as one of the alkaloid toxins. Most of the marine biotoxins have high toxicity. They usually act on some key target sites of the cell membrane such as neural receptors or ion channels [5], causing symptoms such as dizziness, vomiting, diarrhea, muscle pain, heart failure, and even death within a few minutes [6,7,8,9]. For example, TTX at low dose of 0.5~3 mg can make an adult die of poisoning, because TTX can block Na^+^ channels on nerve cell membranes selectively and further leads to nerve paralysis, respiratory failure, and finally death [10,11,12].

Achieving specific and sensitive screening is the most effective way to prevent emergent issues caused by marine biotoxins; however, the previous detection methods cannot meet the requirements because of ethical or technical drawbacks. Cytotoxicity-based mouse bioassay is standardized for testing overall marine toxin toxicity [13], according to the survival time of mice and the poisoning symptoms after injection; however, the mouse bioassay receives much ethical criticism and has technical drawbacks, including poor specificity, high cost, and high variability [14,15,16,17,18]. Some other alternative methods, such as thin-layer chromatography and phosphatase activity inhibition are easy to be operated; however, these methods exhibit low specificity and limited applicability [19,20,21,22]. High performance liquid chromatography (HPLC) and HPLC-mass spectrometry (HPLC-MS) are believed to be interchangeable with mouse bioassay [23,24,25,26,27,28], as HPLC and HPLC-MS can distinguish the toxin structure accurately and achieve accurate quantitation; however, these methods require professional instrumentation and the analysis process is time-consuming, not suitable for *on-site* monitoring. Enzyme linked immunosorbent assay (ELISA) and other antibody-related immunosensors then attract much interest because of the high specificity based on the specific antibody-antigen interaction [29,30,31,32,33,34]; however, these methods have limited availability. Because many marine biotoxins are low weight molecules and have high toxicity, the antibody production needs complicated steps and high cost. Additionally, antibodies are easily denatured and thus may result in the low repeatability of the detection methods. Given the varieties of drawbacks of these previous analytical methods, it is urgent and significant to explore novel recognition elements and detection methods, which can achieve rapid, cost effective, highly specific, and highly sensitive screening.

Recently, aptamers and aptasensors emerged as novel and potential tools for rapid detection [35,36,37,38]. Aptamers can be selected in vitro based on Systematic Evolution of Ligands by Exponential Enrichment (SELEX) [39,40], with high affinity and specificity and with no limitation to the target size [41,42]. The highly specific binding between aptamers and the targets are mainly based on adaptive folding of aptamers under specific ion condition to form specific three-dimensional structures containing hair folds, pseudoknots, convex rings, G-quartets, etc. [38,43]. Compared with antibodies, aptamers show significant advantages in terms of low generation cost, quick chemical synthesis, and the excellent batch unity. Moreover, aptamers have similar or even higher specificity than antibodies [44,45,46], for example, the aptamers can even distinguish chiral molecules and analogs that have a little structural difference, such as the L and D-amino acid [46,47]. Aptamers are easily labeled and fabricated into various aptasensors to achieve rapid, sensitive, and specific detection [48,49,50]. In recent years, an increasing number of highly specific aptamers and highly sensitive aptasensors have been reported. The selected aptamer shows high affinity and specificity, and most of the developed aptasensors are simple to be performed with miniaturized instruments to achieve *on-site* monitoring of marine biotoxins. The advances of aptamers and aptasensors greatly promote the development of marine biotoxins detection, and thus it is necessary to provide a summary of the recent advances.

In this review, we summarized the aptamer-related advances for marine biotoxin to provide a comprehensive summary of aptamer selection and aptasensor development for marine biotoxins, including the detailed information of each aptamer and each kind of aptasensor. All of the reported literatures on aptamer-based research we could find are present herein. Moreover, we forecast the development prospect of aptamers and aptasensors targeting marine biotoxins, based on the existing reports and our own research experience. We hope this review not only provides a comprehensive summary of the recent advances, but also arouses a broad readership amongst academic researchers and industrial chemists. To the best of our knowledge, only two Review papers concerning aptasensors and marine biotoxins have been published so far. In 2018, Bostan et al. summarized a review about optical and electrochemical aptasensors for detection of a kind of marine biotoxin called microcystin-LR (MC-LR) [51]. Only aptasensors for one kind of marine biotoxin, MC-LR, were introduced. Also in 2018, Cunha et al. summarized the aptasensors for aquatic phycotoxins and cyanotoxins [52]. Some aptasensors and aptamers sequences used in the aptasensors were summarized; however, the selection methods of the aptamers, the secondary structure of the aptamers, and the specific ion conditions for aptamer folding were not involved, and many newly published papers about aptasensor development were not included. From the point view of a comprehensive summary of the aptamer-related researches for marine biotoxins, it is better and necessary to provide a more systematic review, which should include the aptamer selection, the detailed information of each aptamer, and each kind of aptasensor.

## 2. Aptamers Targeting Marine Biotoxins

The selection principle of aptamers (SELEX principle) is summarized first. The detailed information of each aptamer is summarized in Table 1. Our summative viewpoints are listed in Section 2.2.

### 2.1. Selection Principle of Aptamers (SELEX Principle)

**Figure 1:**
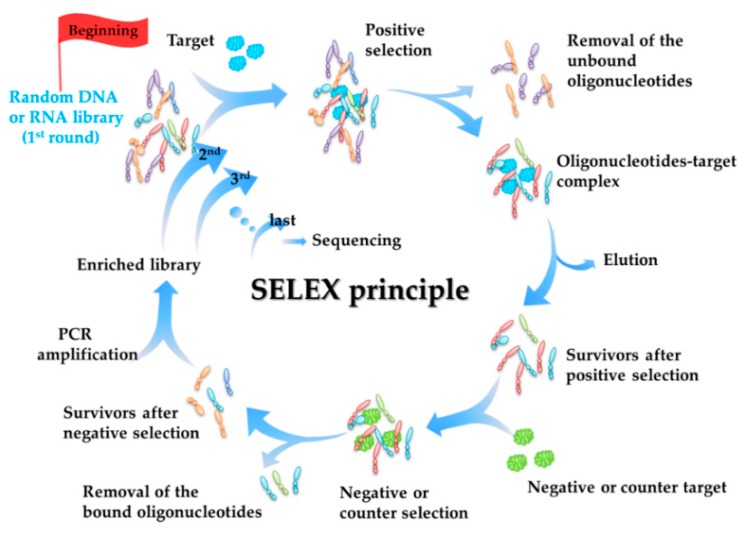
Principle of aptamer selection (SELEX process).

Before summarizing the aptamers that target marine biotoxins, the selection principle of aptamers is introduced herein. The SELEX process is carried out in vitro, with a relatively simple and economical principle [39,40,53]. As shown in Figure 1, the whole SELEX process mainly includes the following parts: (1) Synthesis of a single-stranded oligonucleotide library; (2) Positive selection, including incubation of the library and the targets, partition of the oligonucleotides-target complex and the unbound oligonucleotides, and elution of the bound oligonucleotides from the oligonucleotides-target complex; (3) Negative [54] or counter selection [55], including incubation of the bound oligonucleotides obtained from (2) with negative matrix or analogues of the targets, and partition of the oligonucleotides-target complex and unbound oligonucleotides; (4) PCR amplification of the unbound oligonucleotides obtained from (3); (5) Repetition of the above process so that oligonucleotides that do not bind to the targets or have low affinity to the targets are discarded away gradually, while those having high affinity can be selected with purity increment in each round [35,56]. After several rounds of selection, the last enriched DNA pool is amplified by PCR and sequenced, the affinity of the potential aptamers is analyzed, and the aptamer with highest affinity is finally obtained. Usually, a matrix (X) is needed in the process (2) and (3) to achieve the partition, and the process is thus named as X-SELEX, such as Beads-SELEX [57], Graphene-SELEX (GO-SELEX) [58], and so on.

### 2.2. Detailed Information of Aptamers Targeting for Marine Biotoxins

In recent years, an increasing number of aptamers targeting marine biotoxins have been selected. Detailed database-like information is summarized in Table 1. The target, the name, the selection method, the sequence, and the affinity of each aptamer are listed in detail. Moreover, as the high specific recognition of the aptamers is based on their adaptive folding, we predicted and added the secondary structure of each aptamer in Table 1. The prediction was carried out using an Mfold online server, based on the specific folding condition of each aptamer.

**Figure 2:**
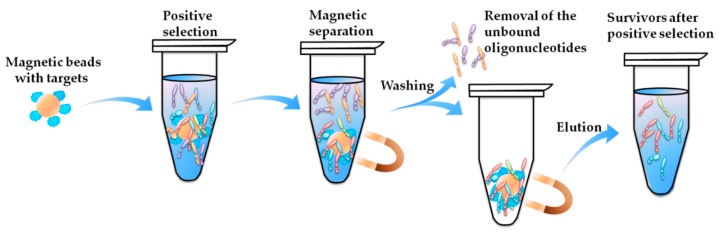
Procedure of positive selection process in Mag-beads-SELEX.

The following are several summative points based the analysis of the literature review:

(a) **Firstly**, aptamers have drawn much attention in the field of marine biotoxins in recent years. According to our investigation, there have been 15 novel aptamers reported, covering all of the three categories of marine biotoxins. Among the targets shown in Table 1, palytoxin (PTX) and okadaic acid (OA) are polyether toxins; brevetoxin-2 (BTX-2) and microcystin (MC) are polypeptide toxins; and tetrodotoxin (TTX), saxitoxin (STX), anatoxin-a (ATX-a), and gonyautoxin1/4 (GTX1/4) are alkaloid toxins. And all of the aptamers were reported after 2012, and 50% of them were successfully selected after 2015. This indicates a high level of academic attention on the aptamers for marine biotoxins.
(b) **Secondly**, most aptamers targeting marine biotoxins were selected using beads-SELEX or magnetic-beads-SELEX (Mag-beads-SELEX), and most of the selection were finished within no more than 20 rounds. The marine biotoxins were immobilized onto the surface of beads or magnetic-beads. The surface with a spherical shape facilitates the full display of the targets on the beads and beads facilitate the convenient separation [59,60]. Figure 2 illustrates the partition and elution process of the positive selection part in the Mag-beads-SELEX. After the incubation of the target-immobilized magnetic beads with the oligonucleotides in the library, the partition of the oligonucleotides-beads complex from the unbound oligonucleotides and the elution of bound oligonucleotides from the oligonucleotides-beads are both achieved using the magnetic separation. The procedure of the beads-SELEX is similar, and the only difference is the partition of the beads and the supernatant is based on centrifugal separation.

Except GTX 1/4, all the remaining targets in Table 1 were immobilized onto beads or magnetic beads for aptamer selection. Gao et al. [61] immobilized the PTX onto Dynabeads^®^M-270 via an amine-carboxylic group coupling as PTX contains a -NH_2_ group. OA, STX, MC-LR, BTX were used as counter-targets in the counter selection. The aptamer with highest affinity, PTX-13, was obtained after 10 rounds. In 2013, Eissa et al. [62] coupled OA onto Diaminodipropylamine agarose beads by coupling the terminal carboxylic groups on OA with the amine groups on the beads via EDC/NHS chemistry. Dinophysistoxin-1 (DTX1)-beads and Dinophysistoxin-1 (DTX2)-beads were used in the counter selection. OA34 was obtained after 18 rounds. In 2015, Eissa et al. [63] immobilized BTX-2 to the divinyl sulfone beads (DVS beads) by coupling the terminal DVS groups on the beads with the hydroxyl groups on BTX-2. Negative DVS beads blocked with ethanolamine were used for negative selection. BT10 was obtained after 10 rounds. Ng et al. [65] selected DNA aptamers for MC-LR, -YR, and -LA. MC-LR, -YR, and -LA were firstly aminoethanethiolled, and then immobilized on NHS-activated sepharose-4B beads. Blank sepharose beads were used in counter selection. AN6, RC4, and HC1 showed high specificity towards MC-LR, MC-LA, and MC-YR, respectively. In 2012, Shao et al. [67] coupled TTX onto acrylic cyclopropane beads for the positive selection and the acrylic cyclopropane beads were used as the negative group. The aptamer was obtained after 10 rounds. Then, in 2014, Shao et al. [68] selected a highly specific aptamer through a combination of beads-SELEX and mutagenic PCR, named A3. Handy et al. [69] conjugated the STX with a protein carrier (keyhole limpet hemocyanin (KLH)) via Jeffamine (2,2′-(ethylenedioxy)bis(ethylamine)) as a spacer based on the Mannich reaction. Then the KLH-STX was coupled on the Dynabeads^®^M-270 epoxy magnetic beads for positive selection. KHL coupled beads were applied as the negative control. APT^STX^ was obtained after 10 rounds. Elshafey et al. [71] conjugated ATX-a to hydrazide modified agarose bead via its terminal carbonyl moiety. Agarose beads were used for negative selections. ATX8 was obtained after 13 rounds. These successful selections of aptamers for marine biotoxins can be referred to for aptamer selection of other marine biotoxins, and maybe most targets can be directly immobilized on beads without any conjugation with protein or other molecules, which is necessary during the antibody generation.

**Figure 3:**
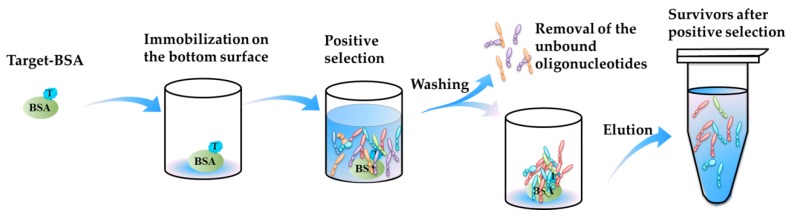
Procedure of positive selection process in Microwell-SELEX. T, target. BSA, bovine serum albumin.

(c) **Thirdly**, some new selection methods promote efficient aptamer selection for marine biotoxins. In 2016, Tian et al. [64] completed a selection of aptamers binding to BTX-2 based on microwell-SELEX. The positive selection process is illustrated in Figure 3. The BTX-2 was coupled with a carrier protein, BSA (bovine serum albumin), and immobilized onto the inner bottom surface of the microwells, and the oligonucleotides were incubated with the immobilized BTX-2. Using the microwells as a matrix, no other special separation instruments were needed for the partition of oligonucleotides-beads complex and the unbound oligonucleotides or the elution of the bound oligonucleotides.

**Figure 4:**
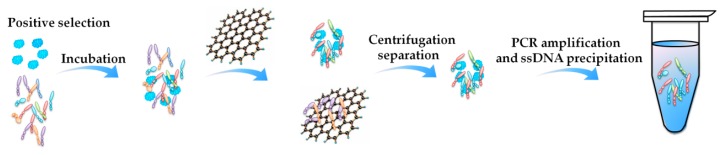
Procedure of positive selection in Graphene-SELEX (GO-SELEX).

In 2016, Gao et al. [72] reported a Graphene-SELEX (GO-SELEX) for selecting aptamer targeting GTX1/4. The small molecule GTX1/4 did not need to be immobilized. As shown in Figure 4, the random ssDNA library was firstly incubated with the target GTX1/4 and then incubated with the GO solution. The ssDNAs that could not bind to the target were absorbed onto the GO surface through π-π stacking and hydrophobic interactions, while the ssDNA that bound to the target remained in the solution with the aptamer-toxin complexes. The GO as well as the absorbed ssDNAs were discarded by centrifugation, while the ssDNA bound to the target in the supernatant was recovered and amplified for the next round selection. An aptamer named as GO18-T was obtained after 8 rounds. Interestingly, the authors performed a Mag-beads-SELEX at the same time, and they found that GO-SELEX exhibited higher efficiency with the following significant advantages. (1) GO-SELEX is easier to be operated without relying on special equipment; (2) Recovery of ssDNA in each round of GO-SELEX was significantly higher than that in Mag-beads-SELEX; (3) Aptamers selected by GO-SELEX exhibited higher affinity in nM range, and the aptamers selected by Mag-beads-SELEX had relatively low affinity ranging from tens of μM to several μM, because the targets were freely distributed in GO-SELEX while they were immobilized in Mag-beads-SELEX, and the immobilization might cause conformational changes to the targets and interference to the binding of the ssDNAs and the conjugation side of the targets. Their practice and analysis showed that GO-SELEX really provided a good reference for aptamer selection for marine biotoxins. If the target does not have any chemical group for immobilization, the GO-SELEX can be chosen.

(d) **Fourthly**, some post optimization greatly improved the affinity of the selected aptamers. Two of the aptamers in Table 1 were derived from truncation study. One is M-30f, and the other is GO-18-T-d. Zheng et al. [70] improved the APT^STX^ selected by Handy et al. [69] to be shorter and to have higher affinity towards STX. The authors analyzed the sequence of APT^STX^ and adopted rational site-directed mutagenesis, on the basis of secondary structure prediction to improve the conformational stability and thus to strengthen its interaction with STX. Then the authors adopted truncation to remove the unnecessary nucleotides and to remain the key binding structure, and M-30f was obtained with a 30-fold improved affinity. The other sample is the GO-18-T-d. It is truncated by Gao et al. [72] after they obtained the GO-18 using GO-SELEX. GO-18 was truncated based on the secondary structure prediction, and the GO-18-T-d has an 8-fold improved affinity.

In short, aptamers targeting marine biotoxins attracts more and more attention, and the recent advances lay a good foundation for further exploration. Different selection methods can be chosen, depending on the individual property of each target. And a post truncation study based on secondary structure prediction can be applied after the successful selection of an aptamer for marine biotoxin.

## 3. Developed Aptasensors Targeting Marine Biotoxins

Aptasensors show great potential in rapid detection. With the development of transducer technology, more and more advanced aptasensors have been reported [73,74,75,76]. For the marine biotoxins detection, there were mainly four kinds of aptasensors reported in recent years, i.e., biolayer interferometry (BLI)-based aptasensors, electrochemistry (EC)-based aptasensors, fluorescence (FL)-based aptasensors, and enzyme linked aptamer assay (ELAA)-based aptasensors. We summarize these four kinds of aptasensors in order, and provide our viewpoints simultaneously. More than 90% of them were reported after 2015. The linear detection range and the limit of detection (LOD) of all these aptasensors were summarized in Table 2 and discussed at the end of this section.

### 3.1. Biolayer Interferometry (BLI)-Based Aptasensors

BLI is a label-free and real-time optical analysis technique that utilizes fiber-optic biosensors for measuring the interactions between biomolecules [77]. The interaction of free analytes with the immobilized ligands on the sensor surface forms a monomolecular layer that in turn creates a proportional shift in the interference spectrum of reflected light (Δλ) [78]. This wavelength shift (Δλ) directly reflects the change in the optical thickness of the sensor layer, and is recorded in real time [79]. There are three BLI-based aptasensors developed for marine biotoxins.

**Figure 5:**
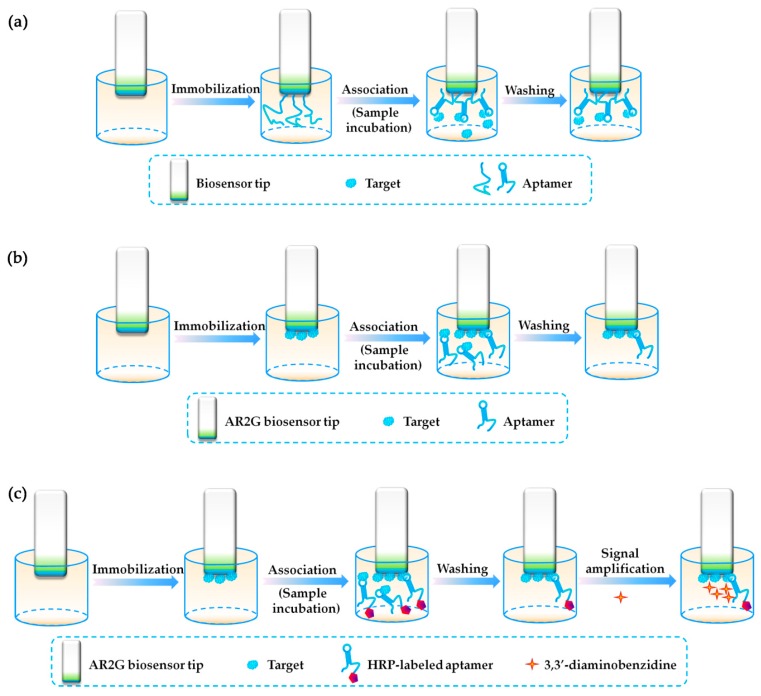
Biolayer Interferometry (BLI)-based aptasensors for marine biotoxin detection. (**a**) Scheme of a label-free BLI-based aptasensor for GTX1/4 detection (Scheme was drawn according to the text description of Ref. [72]); (**b**) Scheme of a label-free and competitive BLI-based aptasensor for STX detection (Scheme was drawn according to the text description and the original Figure 2 of Ref. [80]); (**c**) Scheme of a competitive and signal-amplified BLI-based aptasensor for PTX detection (Scheme was drawn according to the text description and the original Figure 2 of Ref. [61]).

In 2016, Gao et al. [72] used GO18-T-d to construct a label-free and real-time optical BLI aptasensor for the detection of GTX1/4. As shown in Figure 5a, aptamers were immobilized on the sensor tip surface and incubated with the samples so as to capture the target molecules, GTX1/4, in samples. Interference signals were measured after the washing for quantitation. The authors carried out detailed optimization when developing the aptasensor, because the aptasensor signal was significantly dependent on Mg^2+^ concentration and buffer pH. With the optimized working condition, the aptasensor exhibited a high sensitivity and specificity for GTX1/4, when used for detecting GTX1/4 in spiked shellfish samples. A good recovery percentage of 86.70–101.29% was obtained, proving the high accuracy of the aptasensor. Moreover, the aptasensor can be readily regenerated by alkaline denaturation and washing. Then, a label-free and competitive BLI aptasensor for the detection of STX using the M-30f was developed in 2017 by the same group [80]. As shown in Figure 5b, the STX standard was immobilized onto the sensor surface and the aptamer (M-30f) was completely bound by the immobilized STX and the STX in samples. After washing, any change in the number of M-30f bound to the immobilized STX causes a shift in the interference pattern that can be measured in real-time. Similarly, with their previous study [72], they performed detailed working condition optimization for the development of the aptasensor, including the binding time, Na^+^, K^+^ and Mg^2+^ concentrations, and the buffer pH. With the optimized working condition, the developed aptasensor showed high selectivity for STX and good reproducibility and stability, with a good recovery of 101.40–107.26% and a low coefficient of variation of 2.58–6.50%. The excellent practicability of the aptasensor was proven after using three kinds of real samples, including the shellfish matrix, ribbon fish, and the water components. Still in 2017, the same group improved the BLI-based aptasensor design and developed an enzyme-linked competitive BLI-based aptasensor for PTX detection using PTX-13 [61]. As shown in Figure 5c, the target PTX was immobilized on the AR2G surface, and HRP (horseradish peroxidase)-labeled aptamer (PTX-13) was competitively bound by the immobilized PTX and PTX in samples. After washing, the biosensor chip with immobilized PTX: aptamer-HRP complex was submerged in a 3,3’-diaminobenzidine solution and resulted in the formation of a precipitated polymeric product and a large signal change. This design achieved significant great signal amplification, with an excellent LOD at 0.04 pg/mL. The high selectivity of the aptasensor was further verified using the PTX-spiked shellfish and seawater. Although the principle of these three aptasensors is simple, either by a direct capture or a competitive binding similar to ELISA, the BLI is a novel transducer technique in the field of aptamer-based research and it is based on sensitive optical analysis. More efforts can be done to explore more application of the BLI-based aptasensor for marine biotoxin detection.

### 3.2. Electrochemistry (EC)-Based Aptasensor

Electrochemical sensing draws particular attention, owing to its intrinsic advantages in terms of sensitive recognition, rapid response, low cost, excellent portability, modest requirement of sample volumes, and easy signal amplification with redox-active reporters [81,82,83]. Electrochemical aptasensors are very suitable for the rapid and *on-site* detection of marine biotoxins.

**Figure 6:**
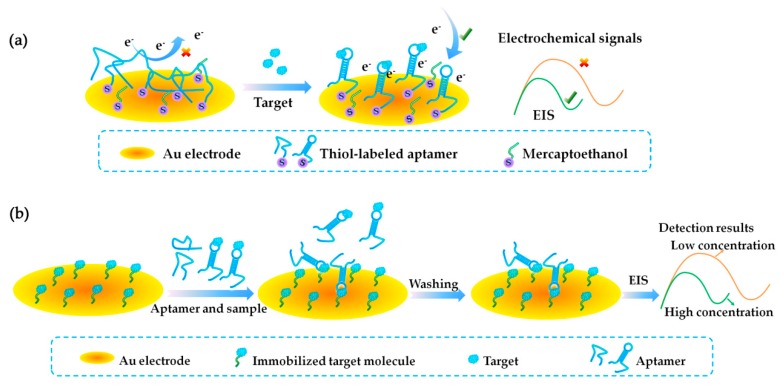
Electrochemistry (EC)-based aptasensors based on gold electrodes. (**a**) Scheme of a label-free EC-based aptasensor for OA detection (Scheme was drawn according to the text description and the original Scheme 1 of Ref. [62]). (**b**) Scheme of a competitive EC-based aptasensor for BTX-2 detection (Scheme was drawn according to the text description and the original Scheme 1 of Ref. [63]). EIS, electrochemical impedance spectroscopy.

In 2013, a label-free EC-based aptasensor was developed by Eissa et al. for OA detection after they selected the anti-OA aptamer, OA34 [62]. The principle is shown in Figure 6a. The –SH-labeled OA34 was immobilized on the Au electrode by a self-assembly approach through an Au−S interaction. With the presence of the target, there will be a significant decrease in the electron-transfer resistance, because the specific binding of the aptamer (OA34) and target (OA) induces an alteration of the aptamer conformation. The electrochemical signals were monitored by electrochemical impedance spectroscopy (EIS), and were negatively correlated with OA concentration. The aptasensor showed high sensitivity and did not show cross-reactivity toward toxins with structures similar to OA such as DTX-1 and DTX-2. The similar scheme was applied to analyze ATX-a using ATX8 in 2015 by Elshafey et al. [71], and their results showed that the aptasensor achieved high sensitivity and high specificity towards ATX-a, with high accuracy and reproducibility. To improve the sensitivity of detection, competitive schemes were further designed. In 2015, Eissa et al. [63] used BT10 to construct a label-free competitive EC aptasensor for BTX-2 detection. The scheme is shown in Figure 6b. The target (BTX-2) standard substance was immobilized on the Au electrode surface via the coupling of its hydroxyl group and the precoated cysteamine, and then the immobilized BTX-2 was incubated with label-free aptamer (BT10) and samples. After washing, the EIS signal was measured. BTX-2 in spiked shellfish extract was analyzed by the aptasensor and a very high recovery percentage (102–110%) was obtained.

**Figure 7:**
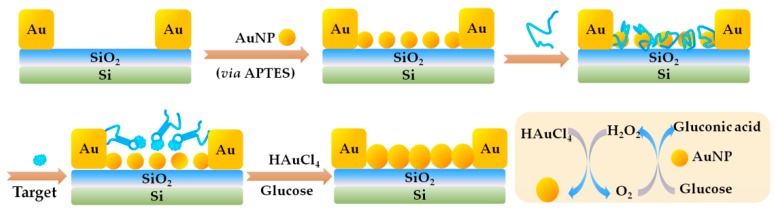
Scheme of a competitive gap-based electrochemical aptasensor for OA detection (Scheme was drawn according to the text description and the original Figure 1 of Ref. [84]).

Another electrochemical aptasensor involving signal amplification was reported in 2017 by Pan et al. [84]. They developed a label-free gap-based electrical competitive aptasensor for OA detection, using the OA34. As is shown in Figure 7, the gap-electrical biosensor is constructed by modifying interdigitated microelectrodes with gold nanoparticles (AuNPs) via APTES ((3-Aminopropyl)-triethoxysilane) mediated electrostatic interaction and using the self-catalytic growth of AuNPs as conductive bridges. The OA aptamer was firstly absorbed onto the surfaces of AuNPs due to electrostatic interaction, and the catalytically active sites of AuNPs are fully blocked. In the presence of OA, the aptamer bound to OA and the AuNPs sites were partially or fully exposed, triggering the catalytic growth in the solution of glucose and HAuCl_4_. The catalytic reaction product H_2_O_2_ in turn reduces HAuCl_4_ to make the AuNPs grow, and thus the conductance signal amplification is closely related to the catalytic activity of AuNPs upon their interaction with the OA and OA aptamer.

**Figure 8:**
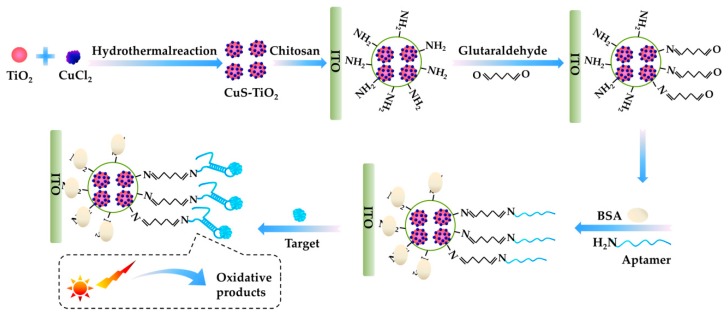
Scheme of a photoelectrochemical aptasensor for detection of microcystin-LR (MC-LR) (Scheme was drawn according to the text description and the original Scheme 1 of Ref. [85]).

In 2018, a photoelectrochemical (PEC) aptasensor was developed by Tang et al. [85] for the detection of MC-LR using the aptamer AN6. As shown in Figure 8, a CuS-TiO_2_ heterojunction composite was prepared by dispersedly depositing CuS nanoparticles on TiO_2_ nanospheres surface with a hydrothermal method, which greatly alleviated the self-aggregation of CuS. The composite exhibited enhanced visible light absorption, and the improved separation of photo-generated charges and the reduced self-aggregation of CuS nanoparticles led to the enhanced photocurrent response. A PEC aptasensor was then constructed by immobilizing CuS-TiO_2_ composite on ITO electrode with chitosan film, which further covalently bounded the aminated aptamer. Glutaraldehyde was used as a linker. When the target MC-LR was captured by the aptamer on the aptasensor, it could be oxidized by the photogenerated hole to impede the electron-hole recombination and further amplify the photocurrent. The PEC aptasensor showed superior analytical performance for MC-LR with a linear range of 5.0 × 10^−5^ nM to 250 nM and a detection limit of 2.0 × 10^−5^ nM. The proposed PEC aptasensor showed high specificity for MC-LR detection in real samples.

The successful development of the above electrochemcial aptasensors proved that it was easy to fabricate aptamers into aptasensors as the aptamers can be easily labelled with thiol group, which can react with the gold electrode or gold nanoparticles. Maybe some other metal particles and nanomaterials can be combined with the electrochemical technique for development of more novel aptasensors to further improve the detection sensitivity. With the development of the electrochemical working station, more and more portable electrochemical aptasensors can be available and can be explored for *on-site* detection of marine biotoxins.

### 3.3. Fluorescence (FL)-Based Aptasensors

Fluorescence-based biosensors also attract much interest, owing to its advantages of high sensitivity, easy labeling, and specific characters [86,87,88]. For example, quantum dots (QDs) has good stability, high molar extinction coefficient, high quantum yield and narrow peak fluorescence emission spectra and other excellent optical properties. Quantum dots have been gradually widely applied as a new aptamer fluorescent marker [89,90]. Different materials were used during the development of FL-based aptasensors for marine biotoxins. There are three studies using up-conversion fluorescence or down-conversion fluorescence, one study using single-walled carbon nanotubes (SWNTs), and two studies using fluorophores.

**Figure 9:**
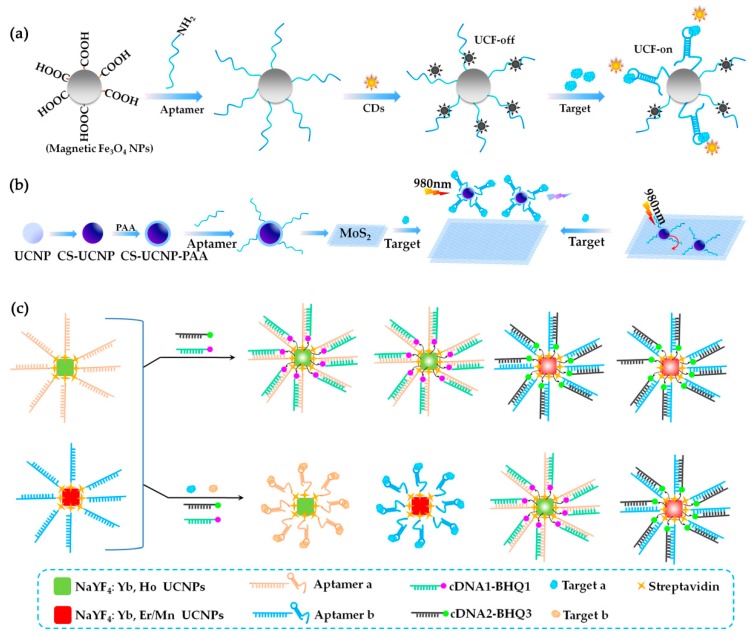
Fluorescence (FL)-based aptasensors using up-conversion fluorescence or down-conversion fluorescence. (**a**) Scheme of a Fe_3_O_4_/aptamer/CDs nanocomposites-based aptasensor for okadaic acid (OA) detection (Scheme was drawn according to the text description and the original Scheme 1 of Ref. [66]). CDs, carbon dots. UCF, up-conversion fluorescence. (**b**) Scheme of a CS-UCNPs and MoS_2_-assisted FL-based aptasensor for MC-LR detection (Scheme was drawn according to the text description and the original Scheme 1 of Ref. [91]). (**c**) Scheme of a dual FRET aptasensor for simultaneous detection of MC-LR and OA (Scheme was drawn according to the text description and the original Figure 1 of Ref. [92]).

In 2017, the aptamer, G11-T, was used by Jin et al. [66] to develop a facilely self-assembled magnetic nanoparticles/aptamer/carbon dots (CDs) nanocomposites for TTX detection. As shown in Figure 9a, thiodiglycolic acid (TA)-stabilized magnetic Fe_3_O_4_ nanoparticles were modified with a NH_2_-labeled aptamer (G11-T), and the CDs then self-assembled on the aptamer to form the Fe_3_O_4_/aptamer/CDs nanocomposites. The nanocomposites exhibited down-conversion fluorescen1ce and up-conversion fluorescence (UCF) emission simultaneously. And the UCF (peaked at 475 nm and excited at 780 nm) increased linearly with the TTX concentration ranging from 0.1 ng/mL to 0.1 mg/mL. Moreover, the author applied more than 10 potential interferences to prove the high selectivity, including toxins (AFB1, AFB2, et al.), biomolecules (histidine, cysteine, et al.), and anions (Cl^−^, PO_4_^3−^ and CO_3_^2−^). Three kinds of real samples were used to prove the high accuracy of the aptasensor, including gastric juice, serum, and urine. In the same year, Lv et al. [91] fabricated an ultrasensitive fluorescence-based aptasensor for detection of MC-LR using the aptamer, AN6. As shown in Figure 9b, the construction of the MC-LR aptasensor was based on an upconversion nanoparticle (CS-UCNPs) and MoS_2_ nanosheets, which have a high affinity toward ssDNA. Aptamer-modified CS-UCNPs were absorbed by MoS_2_ via the van der Waals force between nucleobases and the basal plane of MoS_2_, resulting in the energy transfer from the CS-UCNPs to the MoS_2_ and quenching of the fluorescence. Once MC-LR was added, the aptamer combined with MC-LR preferentially changed the conformation, resulting in the detachment of aptamer-modified CS-UCNPs from MoS_2_ and the reconverment of the fluorescence. The aptasensor provides an ultrasensitive LOD at 0.002 ng/mL and owns well enough reliability and feasibility to allow the determination of MC-LR in real water samples. Another study using upconversion nanoparticles was carried out for the simultaneous detection of more than one kind of toxin. In 2015, Wu et al. [92] reported the first aptasensor for the simultaneous detection of two marine biotoxins, MC-LR and OA. Two aptamers, AN6 and OA34, were used. A homogenous dual fluorescence resonance energy transfer (FRET) system was fabricated between two donor–acceptor pairs. As shown in Figure 9c, green upconversion nanoparticles (NaYF4:Yb, Ho UCNPs) and red upconversion nanoparticles (NaYF4:Yb, Er/Mn UCNPs) were applied as the donors, and BHQ1 and BHQ3 were used as quenchers. The two donor–acceptor couples were fabricated by hybridizing the aptamers with their corresponding complementary DNA. In the presence of MC-LR and OA, the aptamers preferred to bind to their corresponding targets and de-hybridized with the complementary DNA, and thus significantly protected the green and red luminescence from quenching. The upconversion luminescence at 542 and 660 nm was chosen to monitor MC-LR and OA, respectively. The sensitivity of the aptasensor was obtained at pg/mL level. The high specificity was tested using DTX-1, DTX-2, MC-LA, and MC-YR. The practicability of the aptasensor was tested using fish, shrimps, and water samples.

**Figure 10:**
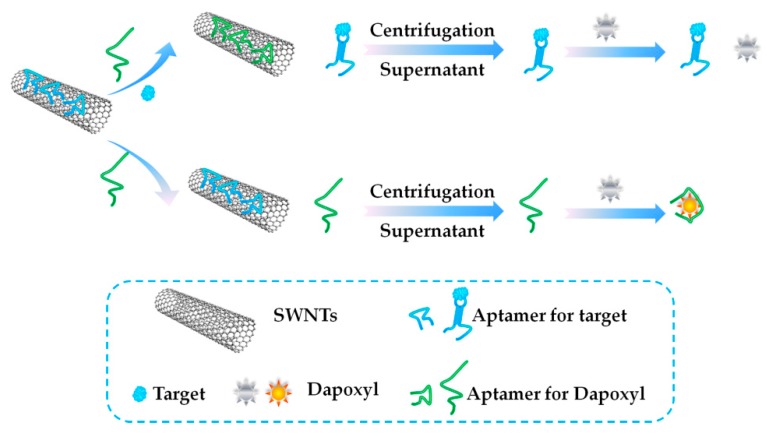
Scheme of a single-walled carbon nanotubes (SWNTs)-assisted fluorescence (FL)-based aptasensor for MC-LR detection (Scheme was drawn according to the text description and the original Scheme 1 of Ref. [93]).

Another nanomaterial, single-walled carbon nanotubes (SWNTs), was used in a FL-based aptasensor constructed by Taghdisi et al. in 2017 [93]. The authors developed a simple fluorescent aptasensor for rapid detection of MC-LR using the aptamer, AN6. SWNTs were used as immobilizers owing to their unique properties of mechanical stability and large surface area. As shown in Figure 10, SWNTs were used as immobilizers, dapoxyl was used as afluorescent dye, DAP-10 was used as a specific aptamer for dapoxyl, and unmodified AN6 was used as a sensing ligand. The aptamer was label-free in this method. In the absence of MC-LR, the dapoxyl could bind to DAP-10, leading to a strong fluorescence intensity. In the presence of MC-LR, DAP-10 bound to the surface of SWNTs, resulting in a very weak fluorescence intensity. What is interesting is that the authors used the dye, dapoxyl. Dapoxyl is a very weak fluorescent dye in water, and usually its fluorescence intensity increases significantly in the presence of organic solvents. Similarly, when the dye binds with its aptamer, DAP-10, its fluorescence intensity is enhanced dramatically because its surrounding environment becomes less polar compared to its environment in water when the dye is free. The authors applied the unique property of the dye and designed the new label-free aptasensor.

**Figure 11:**
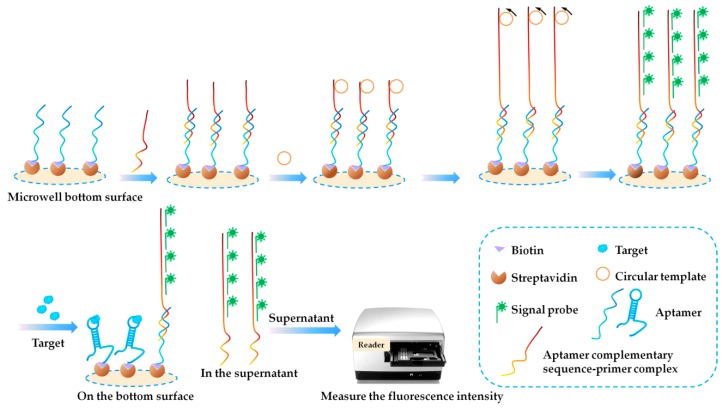
Scheme of a competitive fluorophore-linked aptasensor based on rolling circle amplification (RCA) for okadaic acid (OA) detection (Scheme was drawn according to the text description and the original Figure 1 of Ref. [94]).

Some fluorophores have specific properties and can be applied during the FL-based aptasensor development. In 2015, Alfaro et al. [94] reported the first design of a label-free fluorescent aptasensor for STX detection, using the Evagreen and anti-STX aptamer (APT^STX^). The STX contributed to the secondary structure stability of APT^STX^, resulting in a different double-stranded configuration compared to that of the free aptamer. Increased stability of the aptamer bound to the STX was verified by the distinct melting profiles observed in high resolution melting assay (HRM). Fluorescence from STX-binding aptamers ranged when exposed to different concentrations of STX, and thus the quantitation of STX was achieved. The high specificity of the aptasensor was tested using GTX2/3 as a control. However, the authors found the quantitation correlation fell dramatically when quantifying STX in a rough shellfish extract. This was probably caused by the relatively low affinity of APT^STX^ for STX. In 2017, Gu et al. [95] developed a competitive fluorophore-linked aptamer assay based on rolling circle amplification (RCA) for OA detection using OA34. As shown in Figure 11, biotinylated OA34 was firstly immobilized on the streptavidin coating microwells, and was then hybridized with an aptamer complementary sequence-primer complex. Then RCA reaction was performed to produce a long ssDNA with tandem repeated copies of complementary sequence of the circular DNA template. A large number of FAM (carboxyfluorescein) labeled signal probe was then added to introduce fluorescent signal probes. With the presence of OA in the samples, it would competitively bind with the immobilized aptamer, and led to a large number of detection probes releasing to the supernatant. The supernatant was then collected, and the fluorescence intensity was monitored for quantitation. An excellent LOD at 1 pg/mL was achieved and the aptasensor showed high selectivity towards OA, and almost no interference was observed by DTX-1, DTX-2, STX and DA.

The fluorescence suppliers have specific properties, and aptamers are easily combined with them to develop FL-based aptasensors. Maybe more techniques about nucleic acids amplification can be introduced so as to further improve the sensitivity of FL-based aptasensors.

### 3.4. Enzyme Linked Aptamer Assay (ELAA)-Based Aptasensors

ELISA is a classical analytial method for rapid detection, with the advantages of high sensitivity, easy operation, and high throughput; however, application of ELISA in marine biotoxin detection was limited because of the limited availability of antibodies. Aptamers can be combined with enzyme to develop enzyme-linked aptamer assay (ELAA) for marine biotoxin detection.

**Figure 12:**
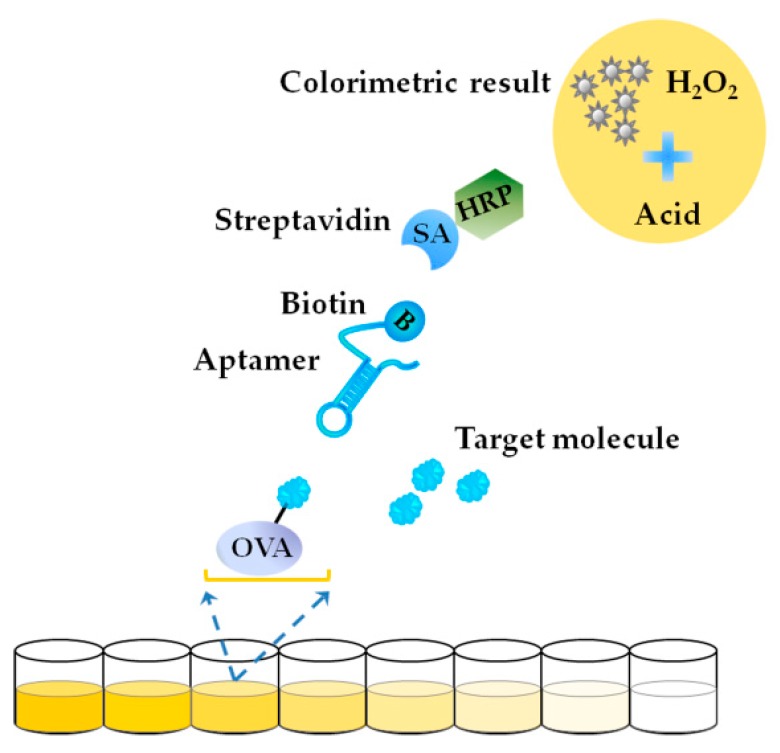
Scheme of an indirect competitive enzyme-linked aptamer assay (ELAA)-based aptasensor for BTX-2 detection (Scheme was drawn according to the text description of Ref. [64]).

In 2016, Tian et al. [64] reported an indirect competitive ELAA-based aptasensor for BTX-2 detection using aptamer Bap5, with the scheme shown in Figure 12. BTX-2-OVA conjugate was immobilized on the bottom of the microwells. A fixed number of biotin-labeled Bap5 was completed by the immobilized BTX-2 and the free BTX-2 in the samples. After washing, horseradish peroxidase (HRP)-conjugated streptavidin was added to introduce the HRP enzyme via the specific binding between streptavidin and biotin. After washing, o-phenylenediamine/H_2_O_2_ solution was added and the enzyme-catalyzed reaction began. The reaction was stopped by the addition of acid. The optical density was measured for quantitation. A LOD of 3.125 ng/mL was obtained, and this sensitivity was greater than that of the NSP (neurotoxic shellfsh poison) ELISA kit for OA (Abraxis, America, Product No. PN520026). ELISA is a kind of typical *on-site* detection method with a commercial absorbance reader and a mature operation protocol, while the production of the antibody of the marine biotoxins is complicated and has high cost. The study herein can be referred for other kinds of marine biotoxins. More aptamers can be used to develop ELAA-based aptasensors for marine biotoxins.

### 3.5. Detailed Information of the Reported Aptasensors for Marine Biotoxin Detection

The above are the recent advances of aptasensors for marine biotoxin detection. For further understanding, we summarized the LOD and linear detection range, as well as other information of the reported aptasensors in Table 2, and obtained the following summative points:

(a) **Firstly**, all of the reported aptasensors achieved high sensitivity, and almost all of them have been validated by real samples. Aptasensors show obvious advantages for sensitive and ultrasensitive detection of marine biotoxins in the real world, compared with the HPLC or MS method. The LODs of the all the reported aptasensors are low enough for the marine biotxins monitoring. LOD of 80% of the reported aptasensors is lower than or equal to 1 ng/mL, LOD of 73% of the aptasensors is lower than or equal to 0.5 ng/mL, LOD of 33% of the aptasensors is lower than or equal to 0.05 ng/mL, and some LOD is even as low as 0.00004 ng/mL. While LOD methods are based on HPLC or MS, they can only achieve LOD at a 1 ng/mL level [23,24,25,26,27,28]. For example, in 2015, Bragg et al. [25] developed an online solid phase extraction hydrophilic interaction liquid chromatography (HILIC) method for the analysis of STX and neosaxitoxin (NEO) in human urine with tandem mass spectrometry, and obtained a LOD at 1.01 ng/mL and 2.62 ng/mL, respectively. A newly reported study in 2018 by Dom et al. [96], which uses liquid chromatography coupled with high resolution mass spectrometry (LC-HRMS) can only achieve a LOD at 1.1~337 ng/g for 18 kinds of marine biotoxins detection. Rey et al. used an improved liquid chromatography coupled with mass spectrometry (LC-MS) for the detection of paralytic shellfish toxins [97]. They tested 15 kinds of biotoxins in four kinds of real samples, and the LOD of the 15 × 14 samples ranged from 0.387 to 55.844 ng/g.
(b) **Secondly**, the sensitivity of the BLI-based aptasensor is relatively higher, showing great advantages in sensitive detection. However, the linear detection range of BLI-based aptasensors was relatively narrow. This may be caused by the limited chip surface space and limited number of immobilized molecules.
(c) **Thirdly**, when compared with other biological alternative methods, the reported aptasensors showed great advantages, as most of the aptasensors achieved LOD below 1 ng/mL. In recent years (from 2014 to now), there are some other alternative methods reported for marine biotoxin detection, such as the cell-based impedance biosensor [98], the SPR (surface plasmon resonance) immunosensor [99], the immunochromatographic sensor [8], and so on. However, most of these alternatives only obtained LOD at about 5 ng/mL. The obvious difference may be due to the higher affinity of the aptamers and the superiority of the aptamers to be easily combined with advanced sensitive transducers.

In short, the development of aptasensors promotes the development of marine biotoxin detection.

## 4. Perspectives

Although there have been many advances in the detection of marine biotoxins, there is still a lot of work that needs to be done. More effort is needed for the further study of aptamer selection, recognition mechanism, and aptasensor development.

(a) **Firstly**, more efforts need to be made to select more aptamers for marine biotoxins. There are only 15 aptamers selected, while there are more than 1000 kinds of marine biotoxins identified in the world. A large number of aptamers is urgently needed. The beads-SELEX and Mag-beads-SELEX can be widely used, referring to the success in the reported selections. The GO-SELEX can be referred, especially for those marine biotoxins that are very small or hard to be immobilized. In addition, some other frontier methods achieve efficient selection [35,100,101,102], such as capillary electrophoresis-SELEX (CE-SELEX) and microfluidic SELEX. CE-SELEX has high-efficiency separation capabilities, and does not need immobilization [103,104,105]. Microfluidic SELEX combines microfluidic chip technology into the aptamer screening process, and can achieve rapid automated selection [106,107].
(b) **Secondly**, the binding mechanism of each marine biotoxin and its aptamer needs to be further studied. Although many aptamers and aptasensors have been developed, the binding mechanism is not clear. So far, most studies concerning aptamer structures stop with Mfold prediction. However, as shown in Table 1, some of the aptamers have one stem and some of them have more than two. Information from secondary structures is not enough for the mechanism study. Further study should be explored, such as the tertiary structure and molecule docking. Only one study concerning the binding format of the aptamer and marine biotoxin was reported. In 2018, Cheng et al. reported their study about binding the way between STX and its aptamer, M-30f [108]. The authors used the circular dichroism spectra, fluorophore and quencher labeled aptamer, and crystal violet based assays to identify the binding way between STX and aptamer. The results show that the conformation of the aptamer is stabilized in PBS buffer (10 mM phosphate buffer, 2.7 mM KCl, 137 mM NaCl, pH 7.4) and K^+^ plays an important role in conformation stability, and this conformation may provide a suitable cave for STX binding. We have been conducting research on the recognition mechanism. We once analyzed the binding between tetracycline and its aptamer [109], using the computational prediction and isothermal titration calorimetry (ITC) experiment. The conformational tertiary structure and the potential binding sites of the aptamer were predicted by computational study and proved by chemical experiment. Our study provides a reference, and other methods [43,89,110] can be further referred.
(c) **Thirdly**, more kinds of aptasensors can be developed. The present aptasensors for marine biotoxins are mainly BLI-based, EC-based, FL-based, and ELAA-based aptasensors. Aptamers show great advantages in terms of easy labeling and easy fabrication. Many other methods can be explored so as to achieve *on-site* detection with high throughput, visual characters, and high portability. In recent years, various aptasensors have been used to detect various kinds of small molecules, such as optical, mass-dependent, lateral flow chromatography-based aptasensors [75,86,111,112,113], and so on. We also developed several kinds of aptasensors for small molecules, such as the indirect competitive [114] and direct competitive [115] ELAA-based aptasensors, AuNPs-based aptasensor, [116] and a SPR-based aptasensor [117]. All of these three kinds of aptasensors performed well for highly sensitive and specific detection. And all of these reported aptasensors can be used to develop more aptasensors for marine biotoxin detection.

In short, future studies should aim to develop new aptamers and new aptasensors so as to strictly monitor the marine biotoxins with highly sensitive and specific methods and to ensure the safety of consumers. Moreover, more attention should be paid to the binding mechanism study so as to understand the fundamental science of the aptamer.

## Figures and Tables

**Table 1:** Detailed information of the aptamers selected for marine biotoxins.

Num.	Target	Aptamer Name	Selection Method	Year	Sequence (5′–3′)	Affinity (Kd, nM)	Secondary Structure	Folding Reference Condition	Ref.
1	Palytoxin (PTX) ^C1^	PTX-13	Mag-beads-SELEX	2017	GGAGGTGGTGGGGACTTTGCTTGTACTGGGCGCCCGGTTGAA	84.3	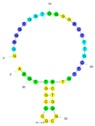	20 mM Tris, 100 mM NaCl, 2 mM MgCl_2_, 5 mM KCl, pH 7.5	[61]
2	Okadaic acid (OA) ^C1^	OA34	Beads-SELEX	2013	GGTCACCAACAACAGGGAGCGCTACGCGAAGGGTCAATGTGACGTCATGCGGATGTGTGG	77	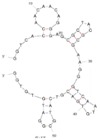	50 mM Tris, 150 mM NaCl, 2 mM MgCl_2_, pH 7.5	[62]
3	Brevetoxin 2 (BTX-2) ^C2^	BT10	Beads-SELEX	2015	GGCCACCAAACCACACCGTCGCAACCGCGAGAACCGAAGTAGTGATCATGTCCCTGCGTG	92	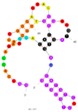	50 mM Tris, 10 mM MgCl_2_, pH 7.5	[63]
4	Brevetoxin 2 (BTX-2) ^C2^	Bap5	Microwell-SELEX	2016	GAGGCAGCACTTCACACGATCTGTGAAGTTTTTGTCATGGTTTGGGGGTGGTAGGGGTGTTGTCTGCGTAATGACTGTAGAGATG	4830	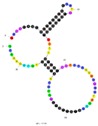	20 mM Hepes, 120 mM NaCl, 5 mM KCl, 1 mM CaCl_2_, 1 mM MgCl_2_	[64]
5	Microcystin-LR (MC-LR) ^C2^	AN6	Beads-SELEX	2012	GGCGCCAAACAGGACCACCATGACAATTACCCATACCACCTCATTATGCCCCATCTCCGC	50	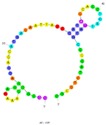	50 mM Tris, 150 mM NaCl, 2 mM MgCl_2_, pH 7.5	[65]
6	Microcystin-LA (MC-LA) ^C2^	RC4	Beads-SELEX	2012	CACGCACAGAAGACACCTACAGGGCCAGATCACAATCGGTTAGTGAACTCGTACGGCGCG	76	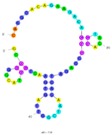	50 mM Tris, 150 mM NaCl, 2 mM MgCl_2_, pH 7.5	[65]
7	Microcystin-YR (MC-YR) ^C2^	HC1	Beads-SELEX	2012	GGACAACATAGGAAAAAGGCTCTGCTACCGGATCCCTGTTGTATGGGCATATCTGTTGAT	193	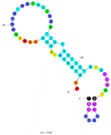	50 mM Tris, 150 mM NaCl, 2 mM MgCl_2_, pH 7.5	[65]
9	Tetrodotoxin (TTX) ^C3^	G11-T ^⁑^	Truncation	2012	AAAAATTTCACACGGGTGCCTCGGCTGTCC	N/A	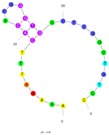	250 mM NaCl, 1 mM MgCl_2_, 0.1 mM EDTA, 1 mM DTT, 20 mM Tris-HCl, pH 7.5	[66,67]
10	Tetrodotoxin (TTX) ^C3^	A3	Beads-SELEX	2014	GGGAGCTCAGAATAA ACGCTCAACCCTGCCGGGGGCTTCTCCTTGCTGCTCTGCTCTGTTCGACATGAGGCCCGGATC	N/A	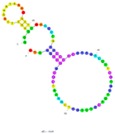	10 mM PBS, pH 7.5	[68]
11	Saxitoxin (STX) ^C3^	APT^STX^	Mag-beads-SELEX	2013	GGTATTGAGGGTCGCATCCCGTGGAAACATGTTCATTGG GCGCACTCCGCTTTCTGTAGATGGCTCTAACTCTCCTCT	3840	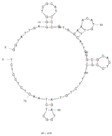	10 mM phosphate buffer, 2.7 mM KCl, 140 mM NaCl, 0.05% Tween-20, pH 7.4	[69]
12	Saxitoxin(STX) ^C3^	M-30f	Truncation	2015	TTGAGGGTCGCATCCCGTGGAAACAGGTTCATTG	133	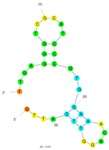	10 mM phosphate buffer, 2.7 mM KCl, 140 mM NaCl, 0.05% Tween-20, pH 7.4	[70]
13	Anatoxin-a (ATX-a) ^C3^	ATX8	Beads-SELEX	2015	TGGCGACAAGAAGACGTACAAACACGCACCAGGCCGGAGTGGAGTATTCTGAGGTCGG	81.378	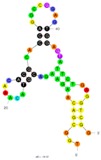	50 mM Tris, pH 7.5, 150 mM NaCl, 2 mM MgCl_2_, pH 7.5	[71]
14	Gonyautoxin1/4 (GTX1/4) ^C3^	GO18-T	GO-SELEX	2016	AGCAGCACAGAGGTCAGATGCAATCGGAACGAGTAACCTTTGGTCGGGCAAGGTAGGTTGCCTATGCGTGCTACCGTGAA	62	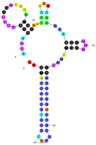	20 mM Tris-HCl, 100 mM NaCl, 2 mM MgCl_2_, 5 mM KCl, pH 7.5	[72]
15	Gonyautoxin1/4 (GTX1/4) ^C3^	GO18-T-d	Truncation	2016	AACCTTTGGTCGGGCAAGGTAGGTT	8.1	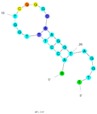	20 mM Tris–HCl and 10 mM MgCl_2_, pH 7.5	[72]

Num., number; Ref., reference; N/A, not available; ^⁑^ The aptamer was named as G11-T in this review, because several papers cited the sequence but it was not wholly consist with that in the original selection paper; ^C1^, polyether toxins; ^C2^, polypeptide toxins; ^C3^, alkaloid toxins. Secondary structure of each aptamer was predicted using the Mfold online sever, with the parameters listed in the “Folding reference condition”.

**Table 2:** Detailed information of the developed aptasensors for marine biotoxin detection.

Target	Aptamer	Aptasensor	Year	Linear detection Range (ng/mL) ^a^	LOD (ng/mL) ^a^	Samples	References
GTX1/4	GO18-T-d	BLI-based	2016	0.2~200	0.05	shellfish	[72]
STX	M-30f	BLI-based	2017	0.1~0.8	0.5	shellfish	[80]
PTX	PTX-13	BLI-based	2017	0.2~0.7	0.00004	shellfish, seawater	[61]
OA	OA34	EC-based	2013	0.1~60	0.07	shellfish	[62]
ATX	ATX8	EC-based	2015	1~100	0.5	drinking water, certified samples	[71]
BTX-2	BT10	EC-based	2015	0.01~2000	0.106	shellfish, mussel	[63]
OA	OA34	EC-based	2017	5~100	1	buffer	[84]
MC-LR	AN6	EC-based	2018	5.0 × 10^−5^~248.8	2.0	water	[85]
TTX	G11-T	FL-based	2017	0.1~100,000	0.06	fish	[66]
MC-LR	AN6	FL-based	2017	0.01~50	0.002	water	[91]
MC-LR and OA	AN6 for MC-LR and OA34 for OA	FL-based	2015	0.1~50	0.025 for MC-LR and 0.05 for OA	water, shrimps, fish	[92]
MC-LR	AN6	FL-based	2017	0.4~1194	0.137	water, serum samples	[93]
STX	APT^STX^	FL-based	2015	15~3000	7.5	gastric juice, serum, urine	[94]
OA	OA34	FL-based	2017	0.001~100	0.001	shellfish	[95]
BTX-2	Bap5	ELAA-based	2016	3.125~200	3.125	buffer	[64]

^a^ converted to be ng/mL for easy comparison. LOD, limit of detection.
